# The WATCHMAN Device Is a Promising Solution for Stroke Prevention in Non-Valvular Atrial Fibrillation: Challenges and Future Directions in South and Southeast Asia

**DOI:** 10.7759/cureus.84677

**Published:** 2025-05-23

**Authors:** Raymond Haward, Joshua Chacko, Kiran K Dhivakaran, Shankar Biswas, Rachel Haward

**Affiliations:** 1 Internal Medicine, Commonwealth University College of Medicine, Gros Islet, LCA; 2 Internal Medicine, Father Muller Medical College, Mangalore, KNA; 3 General Medicine, Sri Muthukumaran Medical College Hospital and Research Institute, Chennai, IND; 4 Medicine, Ivano-Frankivsk National Medical University, Ivano-Frankivsk, UKR; 5 Internal Medicine, Kurunji Venkatramana Gowda (KVG) Medical College & Hospital, Sullia, IND

**Keywords:** anticoagulation therapy, left atrial appendage occlusion, non-valvular atrial fibrillation, stroke prevention, watchman device

## Abstract

The WATCHMAN device (Boston Scientific Corporation, Marlborough, USA), a left atrial appendage occlusion (LAAO) device, represents a significant advancement in stroke prevention for patients with non-valvular atrial fibrillation (AFib) who are contraindicated for long-term anticoagulation therapy. By sealing the left atrial appendage (LAA), the device reduces the risk of thromboembolic events, offering an alternative to traditional anticoagulants like warfarin and direct oral anticoagulants (DOACs). Despite its proven efficacy in clinical trials, such as perforation-reducing outcomes for transcatheter endocardial closing technology (PROTECT-AF) and proactive risk evaluation validation and integrated lifecycle (PREVAIL), the adoption of the WATCHMAN device in South and Southeast Asia faces significant challenges. High procedural costs, limited healthcare infrastructure, inadequate insurance coverage, and a lack of specialized training for interventional cardiologists hinder its widespread use. Additionally, the prevalence of AFib in these regions, though lower than in Western populations, is rising, necessitating innovative solutions for stroke prevention. This article explores the barriers to WATCHMAN Device adoption in South and Southeast Asia, including cost, accessibility, and awareness, while proposing future directions such as local manufacturing, government subsidies, and enhanced training programs. Multicenter studies, public awareness campaigns, and collaboration between healthcare stakeholders are essential to improve access and outcomes. By addressing these challenges, the WATCHMAN device can play a pivotal role in reducing stroke risk and improving the quality of life for AFib patients in these regions.

## Editorial

Understanding the WATCHMAN device

The WATCHMAN device (Boston Scientific Corporation, Marlborough, USA) is a left atrial appendage occlusion (LAAO) device designed to reduce the risk of stroke in patients with non-valvular atrial fibrillation (AFib) who are contraindicated for long-term anticoagulation therapy, such as warfarin or direct oral anticoagulants (DOACs) [1]. This self-expanding implant is deployed in the left atrial appendage (LAA) of the heart, where it acts as a physical barrier to prevent thrombus formation. By sealing off the left atrial appendage, the device mitigates the risk of embolic stroke originating from the appendage [2].

The WATCHMAN device is specifically indicated for non-valvular AFib, as the irregular blood flow in the LAA during AFib predisposes patients to blood stasis and subsequent clot formation [1]. By occluding the LAA, the device prevents thrombi from entering the systemic circulation, thereby reducing the risk of embolic events such as stroke or organ infarction [2]. However, the device is not recommended for use in valvular AFib due to the inherent thrombogenic nature of mechanical heart valves, which can still lead to clot formation despite LAA occlusion [3].

**Figure 1:**
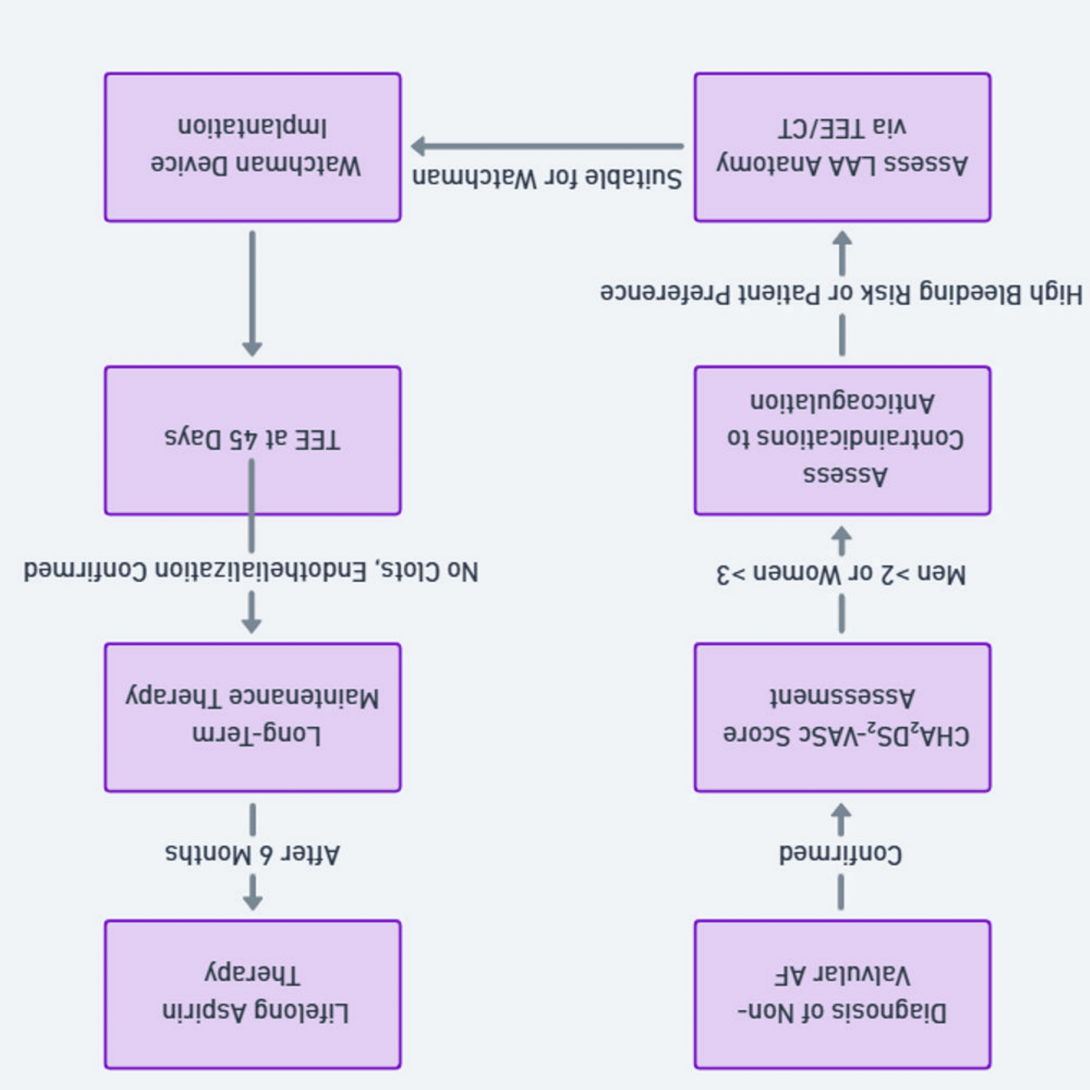
Patient selection and post-procedure care for WATCHMAN device AF: Atrial fibrillation; CHA₂DS₂-VASc: Congestive heart failure, Hypertension, Age ≥75 years (2 points), Diabetes mellitus, prior Stroke or TIA or thromboembolism (2 points), Vascular disease, Age 65–74 years, Sex category (female); LAA: Left atrial appendage; TEE: Transesophageal echocardiography; CT: Computed tomography.

Clinical trials, including PROTECT-AF and PREVAIL, have demonstrated the safety and efficacy of the WATCHMAN device in patients with non-valvular AFib [1,2]. These studies contributed to the device's approval by the U.S. Food and Drug Administration (FDA) for stroke prevention in this patient population [4]. Table 1 delineates the inclusion and exclusion criteria for the use of the WATCHMAN device [1,2,4-6]. The indication for the WATCHMAN device begins with a diagnosis of non-valvular atrial fibrillation, followed by an assessment of a CHA₂DS₂-VASc score >2. Patients must also have a contraindication to long-term anticoagulation due to a high risk of bleeding due to medication intolerance (Figure 1). Subsequently, the left atrial anatomy is evaluated using transesophageal echocardiography (TEE) or cardiac CT imaging to determine the suitability for WATCHMAN implantation [1,2,7]. After device placement, short-term anticoagulation is required to facilitate the endothelization of the device, a process that typically takes approximately 45 days. During this period, the device surface may pose a risk for clot formation. Once endothelization is complete, the left atrial appendage is effectively sealed, preventing clot formation originating from the left atrium [1,8]. Post the 45-day period, the patient’s transition to dual antiplatelet therapy, typically aspirin and clopidogrel, is typically for six months. After this, they can shift to single antiplatelet therapy, such as aspirin, which carries a lower bleeding risk compared to long-term anticoagulation [5,7].

**Table 1:** Inclusion and exclusion criteria for WATCHMAN implantation AFib: Atrial fibrillation; CHA₂DS₂-VASc: Congestive heart failure, Hypertension, Age ≥75 years (2 points), Diabetes mellitus, prior Stroke or TIA or thromboembolism (2 points), Vascular disease, Age 65–74 years, Sex category (female); DOACs: Direct oral anticoagulants; LAA: Left atrial appendage; TEE: Transesophageal echocardiography; CT: Computed tomography; HAS-BLED: Hypertension, Abnormal renal/liver function, Stroke, Bleeding history or predisposition, Labile INR, Elderly (age >65), Drugs/alcohol.

Category	Inclusion Criteria	Exclusion Criteria
Clinical Diagnosis	Non-valvular atrial fibrillation (AFib)	Valvular AFib (e.g., mitral valve stenosis or mechanical heart valve)
Stroke Risk	CHA₂DS₂-VASc score ≥ 2 (moderate to high stroke risk)	CHA₂DS₂-VASc score < 2 (low stroke risk)
Anticoagulation	Eligible for short-term anticoagulation (e.g., warfarin or DOACs) post-implant	Contraindication to both short-term and long-term anticoagulation
Age	Typically, ≥ 18 years old	Pediatric patients or those with life expectancy < 1 year
Anatomy	LAA anatomy suitable for WATCHMAN Device implantation (assessed via imaging, e.g., TEE or CT)	LAA anatomy unsuitable for WATCHMAN Device implantation
	Pediatric patients or those with life expectancy < 1 year	
Bleeding Risk	High risk of bleeding (e.g., HAS-BLED score ≥ 3) or contraindication to long-term anticoagulation	Active bleeding or high risk of bleeding that precludes short-term anticoagulation post-implant
Other Conditions	Willing and able to comply with post-procedure follow-up and antiplatelet therapy	Active infection, endocarditis, or unresolved pericardial effusion

Barriers to adoption in South and Southeast Asian countries

The USA and European nations have actively implemented the WATCHMAN device for their patients, while in South and Southeast Asia, only Singapore has widely adopted this procedure within its healthcare system [9]. When it comes to South and Southeast Asian nations, the challenge is the expense of this procedure. In India, this procedure, although cheaper than in developed countries, can cost 9000 to 12000 USD, making it challenging to afford for a broad patient base. Most countries in this region have out-of-pocket healthcare models with poor insurance coverage [10]. Since warfarin and the INR test are significantly cheaper, easily available, and widely used with good data in these regions, the familiarity and low cost make it a real-world picture alternative [11]. Contraindications to warfarin, such as a history of major bleeding, liver disease with coagulopathy, frequent falls, poor INR control, and medication intolerance, underscore the need for alternative stroke prevention strategies like the WATCHMAN device, which offers a safer option by occluding the left atrial appendage without the risks associated with anticoagulation [12].

Another obstacle in South and Southeast Asian countries is the lack of enough trained specialists who can perform WATCHMAN device implantation, especially in small cities and rural areas where catheterization laboratories as well as electrophysiologists are not easily accessible [13].

Another limitation is the conservative treatment mindset of some physicians who are worried about the postoperative complications like device embolization and pericardial effusion [14].

In India, for example, WATCHMAN devices were introduced in 2017, but there is slow adoption into widespread usage [15]. Besides, atrial fibrillation is more prevalent in White patients than in other racial and ethnic groups [16]. There are alternative devices like Amplatzer Amulet and Lambre that are cheaper and can be considered for cost-effectiveness [17]. Next-generation devices like WATCHMAN FLX have improved sealing against the left atrial appendage, and it is equally important to update such treatments globally to improve patient comfort [18]. Even artificial intelligence-driven transesophageal echocardiography (TEE) interpretation for better case selection will be implemented [19].

Future directions

We urgently need multicenter studies in South and Southeast Asian populations that compare the rates of atrial fibrillation, the safety of the procedure, and the results, as well as WATCHMAN vs. DOACs. Local manufacturing of left atrium occlusion devices can reduce costs significantly [20]. Government subsidies and insurance coverage are required to adopt the WATCHMAN implantation technique into healthcare systems [21]. Establishing more training centers for interventional cardiologists in teaching hospitals, along with expanding TEE training, is crucial for enhancing procedural success, versatility, and skill. Increase TEE (transesophageal echocardiography) training to improve procedural success. Collaborate with global medical device companies to provide hands-on workshops. Another solution is to conduct medical conferences and continuing medical education (CME) programs to educate doctors on WATCHMAN’s benefits [22]. The policymakers should also implement public awareness initiatives to educate high-risk AF patients about stroke prevention options. Encourage shared decision-making between doctors and patients. Also, there should be collaboration between cardiology societies and government health bodies to create funding mechanisms.

Conclusion

The WATCHMAN device represents a transformative advancement in stroke prevention for atrial fibrillation patients, particularly in regions like South and Southeast Asia, where healthcare disparities and resource limitations pose significant challenges. By addressing cost barriers such as local manufacturing and insurance coverage, enhancing training, generating regional data, and fostering collaboration, these regions can unlock the full potential of WATCHMAN devices. A concerted effort by governments, healthcare providers, and industry stakeholders will be essential to ensure equitable access and improved outcomes for patients at risk of atrial fibrillation-related stroke. The future of stroke prevention in South and Southeast Asia lies in innovation, education, and inclusive healthcare policies that prioritize patient well-being.
